# Synergistic Radiosensitization by Gold Nanoparticles and the Histone Deacetylase Inhibitor SAHA in 2D and 3D Cancer Cell Cultures

**DOI:** 10.3390/nano10010158

**Published:** 2020-01-16

**Authors:** Nóra Igaz, Krisztina Szőke, Dávid Kovács, Andrea Buhala, Zoltán Varga, Péter Bélteky, Zsolt Rázga, László Tiszlavicz, Csaba Vizler, Katalin Hideghéty, Zoltán Kónya, Mónika Kiricsi

**Affiliations:** 1 Department of Biochemistry and Molecular Biology, University of Szeged, Közép fasor 52, H-6726 Szeged, Hungary; noraigaz@gmail.com (N.I.); krisztajung@gmail.com (K.S.); kvcs.david@gmail.com (D.K.); 2 Doctoral School of Biology, University of Szeged, Közép fasor 52, H-6726 Szeged, Hungary; 3 Institute of Biochemistry, Biological Research Centre, Szeged, Temesvári krt. 62, H-6726 Szeged, Hungary; andi.buhala@gmail.com (A.B.); vizler.csaba@brc.hu (C.V.); 4 Department of Oncotherapy, University of Szeged, Korányi fasor 12, H-6720 Szeged, Hungary; makika8@freemail.hu (Z.V.); katalin.hideghety@gmail.com (K.H.); 5 Department of Applied and Environmental Chemistry, University of Szeged, Rerrich Béla tér 1, H-6720 Szeged, Hungary; peti0225@gmail.com (P.B.); konya@chem.u-szeged.hu (Z.K.); 6 Department of Pathology, University of Szeged, Állomás utca 2, H-6720 Szeged, Hungary; razga.zsolt@med.u-szeged.hu (Z.R.); tiszlavicz.laszlo@med.u-szeged.hu (L.T.)

**Keywords:** radiosensitization, gold nanoparticles, histone deacetylase inhibitor, combinational treatment, DNA damage, 3D cell culture

## Abstract

Radiosensitizing agents are capable of augmenting the damage of ionizing radiation preferentially on cancer cells, thereby increasing the potency and the specificity of radiotherapy. Metal-based nanoparticles have recently gathered ground in radio-enhancement applications, owing to their exceptional competence in amplifying the cell-killing effects of irradiation. Our aim was to examine the radiosensitizing performance of gold nanoparticles (AuNPs) and the chromatin-modifying histone deacetylase inhibitor suberoylanilide hydroxamic acid (SAHA) alone and in combination. We observed that the colony-forming capability of cancer cells decreased significantly and the DNA damage, detected by γH2AX immunostaining, was substantially greater after combinational treatments than upon individual drug exposures followed by irradiation. Synergistic radiosensitizing effects of AuNPs and SAHA were proven on various cell lines, including radioresistant A549 and DU-145 cancer cells. 3D cultures often manifest radio- and drug-resistance, nevertheless, AuNPs in combination with SAHA could effectively enhance the potency of irradiation as the number of viable cells decreased significantly when spheroids received AuNP + SAHA prior to radiotherapy. Our results imply that a relaxed chromatin structure induced by SAHA renders the DNA of cancerous cells more susceptible to the damaging effects of irradiation-triggered, AuNP-released reactive electrons. This feature of AuNPs should be exploited in multimodal treatment approaches.

## 1. Introduction

In many cases classical chemotherapy leads to only a moderate success rate while provoking a number of unwanted, life-quality-compromising side effects [1]. The efficiency of the treatment is usually increased by the application of chemotherapeutic drug combinations, where each compound exerts a different mode of action, culminating in high-performance cancer cell destruction [2,3]. Another approach to ameliorate patient survival is the simultaneous utilization of different therapeutic strategies, such as radiation therapy, as complementary to the administration of anti-tumor agents [4,5,6,7]. Irradiation, applied with curative or palliative intent, is a common and universally accepted procedure to reduce the size and the metastatic potential of invasive solid tumors. One of the main concerns related to radiotherapy is that it inevitably impacts healthy tissues as well, therefore, novel radiation management modalities are being developed to improve radiation delivery without compromising the structure and the physiology of healthy tissues [8]. It is well known that the administration of radiation causes injury to several cellular components directly, or indirectly via free-radical production. Among cellular targets the DNA and various membrane structures are the most vulnerable to the damaging effects of irradiation [9,10]. Therefore, preventing completely or at least diminishing the injury of non-cancerous tissues caused by high-dose irradiation provides much of the impetus for current attempts to minimize the applied dose of radiotherapy. As a consequence, in recent years several studies highlighted the advantages of utilizing radiosensitizers in tumor therapy [11]. Administration of radiosensitizing agents—which may or may not have direct anti-cancer effects—augments the efficacy of ionizing radiation on the tumor tissue and at the same time reduces the required dose of the irradiation, thus the injury of the tumor surrounding normal tissues is significantly attenuated [12]. Such enhancement of irradiation efficiency is commonly achieved by the application of chemical elements with high atomic numbers, typically by high-Z metals. For this purpose, gold has been considered as a promising candidate having a high atomic number (Z = 79) and mass energy coefficient compared to soft tissues [13,14].

As nanotechnology gained grounds in medicine, the exploitation of the numerous beneficial physical, chemical and optical properties of metal nanoparticles, especially those of silver, gold and platinum nanosystems seems to be a reasonable approach also in oncotherapy [15,16,17]. A highly favorable feature of nanoparticles, which strongly advocates for their utilization in cancer treatment modalities is their capacity to preferentially accumulate within the tumor tissue due to the enhanced permeability and retention (EPR) effect [18,19]. Apart from EPR, tumor-selective uptake of nanoparticles can be further enhanced via active targeting, since the large specific surface of the particles might serve as an ideal platform for the chemical or physical conjugation of cancer cell-selective ligands [20]. Therefore, metal nanoparticles (especially those with high Z) are able to increase the contrast between soft tissues and the tumor, and at the same time, by exhibiting a radiosensitizing character they enhance the efficiency of the therapy and concomitantly reduce the unwanted side effects [21,22].

Based on the above considerations, gold nanoparticles (AuNPs) hold enormous potential as radiosensitizers in radiation therapy [23]. Apart from the radiation-enhancing feature, further advantageous properties of AuNPs comprise biocompatibility, they are simple to synthesize, easy to modify by the conjugation of various biomolecules and due to their size- and shape-dependent optical, electrical and thermal properties, AuNPs are also suitable for various diagnostic approaches, drug delivery, as well as for therapeutic applications [24,25,26]. All these properties render gold nanoparticles ideal candidates for oncotherapeutic modalities. 

Irradiation of AuNPs triggers physical, chemical and biological events within the material and in its close surroundings. The main physical interactions by which radiation reacts with gold nanoparticles are the photoelectric effect which is usually followed by Auger cascade and the Compton Effect. In both cases, the incident photons cause the ejection of electrons; however, by the photoelectric effect this happens from an inner atomic orbital, causing the outer shell electrons to fall into the place of the vacancy and thereby promoting the release of a cascade of secondary electrons [27]. Which of these interactions dominates depends on the energy of the photon and of the atomic number of the material [28,29,30]. Next to the physical processes, the dose enhancement realized by gold nanoparticles has chemical and biological components as well [22]. From the surface of AuNPs donor electrons are transferred to oxygen molecules leading to the rapid generation of free oxygen radicals, which will ultimately react with mitochondria and other membrane structures and with the DNA, resulting eventually in DNA single-strand or double-strand breaks. Failure of the repair mechanisms leaves the radiation-induced damage uncorrected, causing cell cycle arrest and triggering apoptotic or necrotic cell death after the radiation [31].

Besides the novel nanoparticle radiosensitizers, many clinically used anti-cancer agents possess radiation enhancing activity. As an example, it has been shown that some histone deacetylase (HDAC) inhibitors like Vorinostat (also known as suberoylanilide hydroxamic acid, SAHA), applied in the clinical practice as a potent antineoplastic agent against different types of leukemia and solid tumors [32], might function effectively as radiosensitizer, broadening the spectrum of their utilization in different treatment modalities [33,34]. 

Indeed, HDAC inhibitors have been employed in cancer therapy based on the fact that inhibition of these enzyme activities—which were largely elevated in several tumor types [35]—can modulate the deviant chromatin structure and the abnormal cytosolic protein functions of the tumors [36,37]. As a matter of fact, protein acetylation, established by lysine acetyl-transferase (KAT or HAT) and removed by lysine deacetylase (KDAC or HDAC) enzyme activities, is an important component of cellular homeostasis [38]. Ultimately, unbalanced activities of these enzymes result in abnormalities of protein functions and cause aberrant chromatin structures [39]. More specifically, inhibition of the HDAC enzymes maintains the N-terminal lysine residues of histone proteins hyperacetylated, resulting in a relaxed chromatin structure, which makes the DNA more vulnerable to various harmful effects, like those induced directly by irradiation or by reactive oxygen species [40]. Additionally, acetylation can influence the stability and the DNA binding affinity of transcription factors (e.g., p53) [41,42,43,44], and alters the regulation of numerous protein families involved in DNA repair, such as Ku and RAD proteins [45,46,47]. Consequently, the histone acetylation-induced opened chromatin structure and the decreased DNA repair capacity are together responsible for the radiation enhancing features of HDAC inhibitors [40,48].

In our previous study, we verified the synergistic interaction between an HDAC inhibitor trichostatin A (TSA) and silver nanoparticles (AgNPs) on cancer cells. We showed that the HDAC inhibitor TSA enhances the DNA targeting activity and apoptosis-inducing efficacy of AgNPs most probably due to their combined effect on chromatin structure, as substantial amounts of reactive oxygen species generated rapidly by reactive silver ions and released from the AgNP surface caused direct damage on supramolecular structures, including the DNA. In cancer cells, treated with TSA, DNA molecules were more accessible to the oxidative stress induced by AgNPs, since the presence of TSA caused the relaxation of the chromatin structure and rendered the DNA more accessible to the action of radicals [49]. Therefore, we concluded that HDAC inhibitors and metal nanoparticles are potentially effective combinational partners in tumor chemotherapy.

In the present work, we aimed to examine whether HDAC inhibition by SAHA and gold nanoparticles are able to potentiate each other’s radiosensitizing features in 2D and 3D cancer cell cultures. To test the combinational radiosensitizing effect of AuNP and SAHA, 2D and 3D cultures of four different cancer cell lines were exposed to AuNP and SAHA, were irradiated and then the subsequent damage caused by chemo- and radiotherapy was detected. We used 3D cell cultures since they encompass much better the cell-cell and cell-extracellular matrix (ECM) interactions formed in an actual tumor than 2D cultures [50,51]. It was previously shown that cells growing in 3D exhibit higher drug- and radioresistance via integrin-mediated processes [52,53,54,55,56], therefore, if a synergistic radiation enhancement by these agents can be established in 3D models, it validates and strengthens the therapeutical applicability of SAHA and gold nanoparticle combinations upon radiotherapy. Therefore, the clear purpose of our study was to apply AuNP and SAHA treatments along with irradiation not only to 2D cancer cell cultures but also on 3D spheroids in order to verify the synergistic radiation enhancing effects of these agents.

## 2. Materials and Methods

### 2.1. Synthesis and Characterization of Gold Nanoparticles (AuNPs)

Quasi-spherical citrate-coated gold nanoparticles (AuNPs) were synthesized according to the modified Lee–Meisel method [57]. Tetrachloroauric acid was reduced by sodium borohydride (0.1%) in the presence of sodium citrate (1%) as stabilizing agent. Morphology and size distribution of the particles obtained were assessed by a FEI Tecnai G^2^ 20 X-Twin (Field Electron and Ion Company (FEI) Corporate Headquarters, Hillsboro, OR, USA) transmission electronmicroscope at an acceleration voltage of 200 kV, furthermore, additional size and surface charge measurements were performed by a Zetasizer Nano ZS instrument (Malvern, Worchestershire, UK). The optical properties of the obtained nanoparticles were assessed by spectral analysis. Absorbance spectra were recorded using an Ocean Optics 355 DH-2000-BAL ultraviolet–visible (UV–Vis) spectrophotometer (Halma PRC, Largo, FL, USA) within the 300–800 nm range.

### 2.2. Cell Culture 

A549 lung adenocarcinoma, DU-145 and PC-3 prostate cancer as well as MCF-7 breast cancer cell lines were purchased from ATCC and maintained in Roswell Park Memorial Institute 1640 (RPMI) medium (Biosera, Metro Manila, Philippines) complemented with 10% Fetal Bovine Serum (FBS) (EuroClone, Pero MI, Italy), 2 mM glutamine (Sigma-Aldrich, St. Louis, MO, USA), 0.01% streptomycin and 0.006% penicillin (Biowest, Nuaille, France). Cells were cultured under standard conditions in a 37 °C incubator at 5% CO_2_ and 95% humidity. 

For 3D cell culture experiments all the solutions were filtered through 0.22 µm membrane filter (Merck, Darmstadt, Germany). U-bottom 96-well plates (Greiner Bio-One, Kremsmünster, Austria) were coated with poly(2-hydroxyethyl methacrylate) (poly-HEMA, Sigma-Aldrich, St. Louis, MO, USA) using 2 times 15 µL of 6 mg/mL poly-HEMA dissolved in 96% ethanol then the plates were left to dry and were UV-sterilized. For spheroid formation 10^4^ cells were seeded into each well of 96-well plates, then cells were centrifuged with 280 g for 5 min and left to grow for a week at 37 °C, 5% CO_2_ and 95% humidity in filtered RPMI medium (Corning, Corning, NY, USA) containing 10% FBS (Gibco, ThermoFisher Scientific, Waltham, MA, USA), 2 mM glutamine, 0.01% streptomycin and 0.006% penicillin.

### 2.3. Electron Microscopy

To visualize the uptake of gold nanoparticles by cancer cells, transmission electron microscope (TEM) images were taken. For this 10^5^ cells were seeded onto 0.4 µm pore size polyester membrane inserts (Corning, Corning, NY, USA). After cells were attached to the membrane, the cultures were incubated with AuNPs in 6.8 µM concentration for 24 h, then cells were washed with Phosphate-Buffered Saline (PBS) and fixed in 4% glutaraldehyde. Samples were first embedded in gelatin, then were sliced into 1–2 mm cubes which were subsequently embedded in epoxy (Embed 812, EMS, PA 19440). To identify the cell monolayer, semi-thin sections of 1 µm were prepared, then thin sections of 70 nm were obtained and stained with uranyl and lead solutions. Images were captured by a Jeol JEM-1400 electron microscope (Jeol Ltd., Tokyo, Japan) using 100 kV voltage. 

### 2.4. Irradiation

The samples were irradiated at the Department of Oncotherapy of the University of Szeged. For irradiation 6 MeV photon beam of a linear accelerator (Primus linear accelerator, Siemens Healthcare GmbH, Erlangen, Germany) was used with source isocenter distance of 100 cm to the cell cultures. Cell culture plates were placed between two 2 cm-thick polymethyl methacrylate slabs to compensate the build-up effect and to ensure homogenous radiation exposure. In every case the isocenters were positioned in the geometrical center of the plates. To maximize the dose homogeneity the half of the planned dose was delivered by a 20 × 20 cm beam downward (gantry angle 0°) while the other half with an identical beam upward (gantry angle 180°).

### 2.5. Viability Assay

To determine the effect of AuNPs and SAHA (MedChemExpress, Monmouth Junction, NJ, USA) with and without irradiation on cancer cell viability, Methylthiazolyldiphenyl-tetrazolium bromide (MTT) assay was performed. For this, 5000 cells/well were seeded from each cell line into 96-well plates (Biologix, Jinan, Shandong, China). On the next day the medium was removed from the cells, the samples were washed with PBS and finally the cells were treated with 6.8 µM; 34 µM; 68 µM of AuNP or 0.1 µM; 0.5 µM; 1 µM of SAHA, and with the combination of AuNP and SAHA in 68:1 constant ratio diluted in cell culture medium containing 10% FBS. After 24 h treatment cells were irradiated with 0 Gy (no radiation treatment) and 2 Gy doses and 48 h later MTT assays were performed according to the following protocol: treatments were removed, cells were washed with PBS and 0.5 mg/mL MTT reagent diluted in RPMI medium was added to the cells for an hour. Formazane crystals were dissolved in dimethyl sulfoxide (DMSO, Serva Electrophoresis GmbH, Heidelberg, Germany) and the absorbance of the samples was measured at 570 nm by Synergy HTX plate reader (BioTek, Biotek Instruments Inc., Winooski, VT, USA). Synergistic, additive or antagonistic effect of combinational drug treatments as well as of 2 Gy irradiation was investigated via combinational index (CI) calculation by CompuSyn Softwer (Version 1.0 by Ting-Chao Chou and Nick Martin). The combinational indices (CI) at effective dose (ED) ED50, ED75, ED90 and ED95 were determined and the average of these values was considered as CI. The data obtained was analyzed by two-way analysis of variance (ANOVA) Tukey’s multiple comparisons test in GraphPad Prism 6 software.

### 2.6. Clonogenic Assay

The radiosensitizing effect of AuNP and SAHA treatments were determined by colony forming assay. For this, 6 × 10^5^ cells/flask were seeded into T25 cell culture flasks (Biologix, Jinan, Shandong, China) and left to grow for 24 h. On the following day cells were treated with 6.8 μM AuNPs or 0.1 μM SAHA or with the combination of 6.8 μM AuNPs and 0.1 μM SAHA. After 24 h treatment, the cells were exposed to 0, 2 or 4 Gy irradiation delivered with a Primus linear accelerator (Siemens Healthcare GmbH, Erlangen, Germany) and were incubated further for 24 h. On the next day cells were trypsinized, suspended in medium and counted. From each sample 700 cells/well were seeded into 6-well plates in 3 replicates and left to grow for 1 week. Then colonies were fixed with a mixture of methanol and acetone (in 7:3 ratio) and stained with 0.5% crystal violet dissolved in 25% methanol. Finally, colonies were counted and from the obtained data the colony forming unit % (CFU %) was determined after normalization to the untreated, non-irradiated control. 

Colony forming assay on cells from the 3D cell cultures was also performed. Spheroids of A549, PC-3 and DU-145 cells were treated either with 34 μM AuNPs or 1 μM SAHA or with their combination, on the other hand, MCF-7 spheroids were exposed to 34 μM AuNP, 0.5 μM SAHA or their combinations for 24 h. Then spheroids were irradiated with 0, 2 or 4 Gy doses (Primus linear accelerator, Siemens Healthcare GmbH, Erlangen, Germany). After 24 h incubation 1-cell suspensions were created from the spheroids using Accumax (Invitrogen, Carlsbad, CA, USA) and 700 cells/well were seeded into 6-well plates from each sample. After 1 week incubation the colonies were fixed in a mixture of methanol and acetone (in 7:3 ratio) and stained with 0.5% crystal violet dissolved in 25% methanol, then colonies were visualized, counted and finally the CFU (%) was determined after normalization of the data obtained to the untreated, non-irradiated control. Statistical evaluation was performed in GraphPad Prism 6 using two-way ANOVA Tukey’s multiple comparisons test.

## 3. Immunocytochemistry

For immunostaining cells were grown on glass cover slips (VWR International, Radnor, PA, USA) placed into 24-well plates (Biologix, Jinan, Shandong, China). To assess histone acetylation level, cells were treated either with 6.8 μM AuNPs, 0.1 μM SAHA or the combination of 6.8 μM AuNPs and 0.1 μM SAHA for 24 h. Samples were washed in PBS and fixed in 4% paraformaldehyde. For permeabilization 0.3% Triton-X-100 (Sigma-Aldrich, St. Louis, MO, USA) solution was used, then the samples were blocked in 5% Bovine Serum Albumin (BSA, Sigma-Aldrich, St. Louis, MO, USA) dissolved is PBS. Cells were incubated with anti-acetylated lysine (Abcam, Cambridge, UK) antibody in 1:200 dilution followed by Dylight 549 fluorophore-conjugated secondary antibody (Abcam, Cambridge, UK) in 1% BSA. Cellular morphology was visualized by the detection of microtubuli using anti-tubulin (Sigma-Aldrich, St. Louis, MO, USA) antibody in 1:600 dilution and anti-mouse Alexa 488 fluorophore-conjugated secondary antibody (Invitrogen, Carlsbad, CA, USA) diluted in 1% BSA. The samples were visualized by OLYMPUS BX51 microscope with Olympus DP70 camera (Olympus, Tokyo, Japan). To quantify the level of acetylated lysine after treatments, fluorescence intensity was also measured by Synergy HTX plate reader (BioTek, Biotek Instruments Inc., Winooski, VT, USA) after immunostaining. 

To assess DNA damage, cells were treated with 6.8 μM AuNPs, 0.1 μM SAHA or their combination (6.8 μM AuNPs + 0.1 μM SAHA) for 24 h. Then cells received 0 or 2 Gy dose irradiation (Primus linear accelerator, Siemens Healthcare GmbH, Erlangen, Germany) and 24 h following the treatment the cells were fixed, permeabilized and blocked using the same procedure as described above. Then, DNA double strand breaks were visualized by staining the samples with anti-γH2AX (Thermo Fisher, Waltham, MA, USA) (1:300) primary antibody followed by Alexa 488 fluorophore-conjugated goat anti-mouse secondary antibody (Invitrogen, Carlsbad, CA, USA) (1:800) diluted in 1% BSA. Cells were washed twice in PBS containing 0.01% Tween-21 (Polyoxyethylene sorbitan monolaurate) and nuclei were stained with Hoechst 33342 (Sigma-Aldrich, St. Louis, MO, USA) solution in 3.25 μM concentration. Samples were examined and the number of the DNA double strand breaks was estimated by Olympus FV10i confocal microscope. The percentage of the γH2AX-positive cells in the samples and the number of γH2AX foci in the positively stained cells were counted by ImageJ software. For statistical analysis, an unpaired *t*-test was used on the obtained data in GraphPad Prism 6.

## 4. Immunohistochemistry

DNA damage from 3D cell cultures was investigated by γH2AX staining. For this, spheroids were fixed in 4% formaldehyde, were embedded into 3% agarose then agarose cubes were embedded in paraffin and 5 mm-thick paraffin slices were cut. After deparaffinization and heat-mediated antigen retrieval DNA double strand breaks were visualized by anti-γH2AX antibody (Thermo Fisher, Waltham, MA, USA) used in 1:300 dilution following horseradish peroxidase-conjugated secondary anti-mouse antibody in 1:600 dilution. Photos were taken under OLYMPUS BX51 microscope with Olympus DP70 camera (Olympus, Tokyo, Japan) and the percentage of γH2AX-positive cells were determined by ImageJ software. For the statistical evaluation unpaired *t*-test was used in GraphPad Prism 6 software.

## 5. Results

### 5.1. Synthesis and Characterization of AuNPs

**Figure 1:**
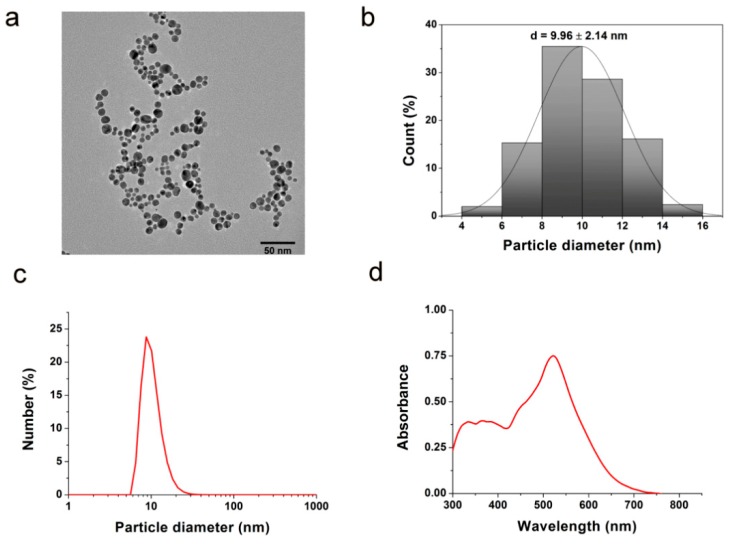
Characterization of gold nanoparticles. (**a**) The chemically synthesized citrate-coated gold nanoparticles (AuNPs) were visualized by transmission electron microscopy. The average diameter of the obtained AuNPs is around 10 nm according to (**b**) transmission electron microscope (TEM) image analysis and (**c**) dynamic light scattering (DLS). (**d**) The characteristic peak around 520 nm on the ultraviolet–visible (UV–Vis) spectrum suggests the presence of AuNPs in the solution.

Gold nanoparticles were prepared by chemical reduction approach and were subsequently characterized by transmission electron microscopy (TEM), dynamic light scattering (DLS) and ultraviolet–visible spectroscopy (UV–Vis). The faceted (quasi-spherical) morphology of the produced AuNPs was verified by TEM (Figure 1a). The average diameter of the obtained nanoparticles proved to be around 10 nm according to TEM image analysis and DLS (Figure 1b,c). Highly negative −36.2 ± 5 mV zeta potential was measured, which indicates negative surface charge of the citrate-coated nanoparticles providing stability for the colloid solution. The characteristic peak around 520 nm on the UV–Vis spectra suggests the presence of gold nanoparticles in the solution (Figure 1d). 

### 5.2. The Internalized AuNPs Do Not Affect the Histone Deacetylase-Inhibiting Activity of Suberoylanilide Hydroxamic Acid (SAHA) in Cancer Cells

**Figure 2:**
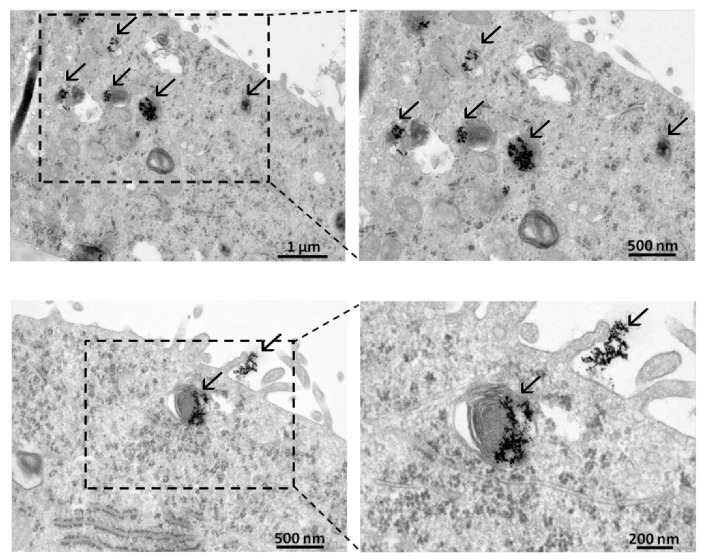
Internalization of AuNPs by A549 adenocarcinoma cells. AuNPs (indicated by arrows) were detected by TEM image analysis on the surface of A549 cells and inside the cytoplasm as well, mostly in multilamellar bodies.

Cancer cells take up gold nanoparticles promptly, as the internalization of AuNPs by A549 lung adenocarcinoma cells was demonstrated by TEM. AuNPs were observed on the surface of the A549 cells and were also detected in the cytoplasm, accumulated mainly in multilamellar bodies (Figure 2).

**Figure 3:**
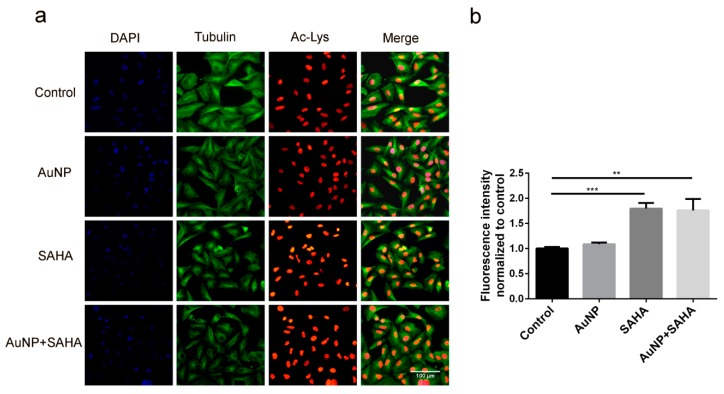
The presence of AuNPs does not affect the histone deacetylase inhibitor activity of SAHA. (**a**) Increased level of acetylated-lysine was observed in the SAHA- and AuNP + SAHA-treated A549 cells, visualized by fluorescence microscopy. (**b**) The fluorescence intensity of acetylated lysine was significantly higher in SAHA- and in AuNP + SAHA-treated samples, quantified by plate reader measurements. (** *p* value = 0.0043; *** *p* value = 0.0003; Unpaired *t*-test).

Since SAHA inhibits histone deacetylase enzymes, the direct consequence of SAHA action is an elevated level of acetylated histones. To prove that SAHA—administered individually or in combination with AuNPs—is capable of exerting its inhibiting effect on HDAC enzymes, we performed immunostaining on A549 cells, which were exposed to 6.8 µM AuNP or to 0.1 µM SAHA alone or to AuNP in combination with SAHA. We detected acetylated lysine levels in the samples by fluorescence microscopy (Figure 3a) and quantified it by plate reader measurements (Figure 3b). The acetylation level upon SAHA and AuNP + SAHA combinational treatments was significantly higher than the fluorescence intensity of the untreated control cells or of those that received only AuNP treatment (Figure 3a,b). Therefore, we concluded that SAHA in combination with AuNPs can in fact accomplish its HDAC inhibiting role and there is no antagonistic effect between AuNPs and SAHA when these two drugs are applied in combination. 

### 5.3. AuNPs and SAHA in Combination Synergistically Decrease Cancer Cell Viability After Irradiation

**Figure 4:**
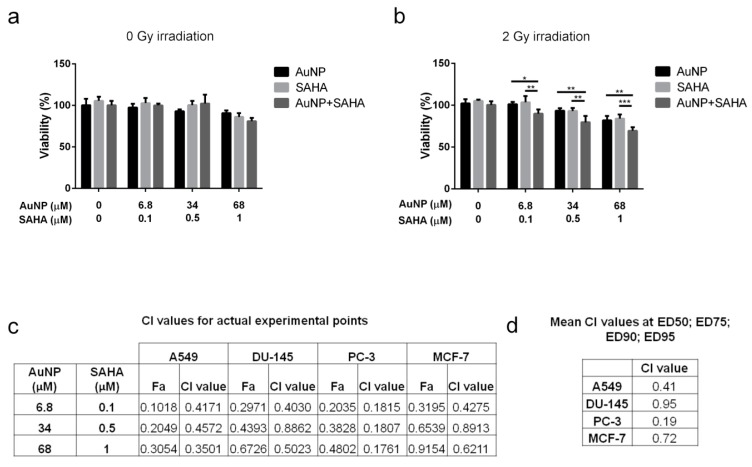
The effect of AuNP, SAHA and the combination of AuNP and SAHA on cell viability: (**a**) without irradiation, no differences in the viability of A549 cells were observed after AuNP, SAHA and AuNP + SAHA treatments. (**b**) After 2 Gy irradiation the viability of A549 cells were significantly decreased in the AuNP + SAHA-treated samples compared to control and to the individual treatments. (* *p* value < 0.05; ** *p* value < 0.01; *** *p* value < 0.001; two-way analysis of variance (ANOVA) Tukey’s multiple comparisons test). (**c**) Combinational indices (CI) for the actual experimental points of the combinational treatments were under 1, suggesting synergism between AuNPs and SAHA in all tested cell lines. (**d**) The mean CI values obtained from ED50, ED75, ED90 and ED95 of A549, DU-145, PC-3 and MCF-7 cell lines indicate synergistic interaction between AuNPs and SAHA in combinational administration.

To examine the nature of the effect of AuNP and SAHA combinational treatments, cancer cells were incubated with either AuNP, SAHA or their combination with or without irradiation and the viability of the cells was determined by MTT assay. The obtained viability data were used to calculate the combinational indices (CI) by CompuSyn software (Version 1.0 by Ting-Chao Chou and Nick Martin) (Figure 4). 

No differences were observed on the viability of samples treated for 72 h with AuNP or SAHA or with the combination of AuNP and SAHA compared to the untreated cells when no irradiation was applied, thus in these cases, no CI was determined (Figure 4a). Cell viability and CI values of A549 cells were assessed after irradiation with 2 Gy dose, since viability was significantly decreased upon AuNP + SAHA treatments compared to the individual exposures after irradiation (Figure 4b). The obtained CI value of AuNP and SAHA on A549 cells was 0.41, suggesting synergism between the two drugs. Strong synergism was detected on PC-3 cells with 0.19 CI value, while lower synergistic interaction was determined on MCF-7 (CI: 0.72) and DU-145 cells (CI: 0.95) (Figure 4d and Supplementary Figure S1). In all cases, the CI values for the actual experimental points were under 1, which indicates that AuNPs and SAHA synergistically enhance each other’s radiosensitizing properties and the observed synergism is general across a panel of cancer lines (Figure 4c).

### 5.4. Combinational Treatments Decrease the Colony Forming Capabilities and Increase the DNA Damage in Cancer Cells

**Figure 5:**
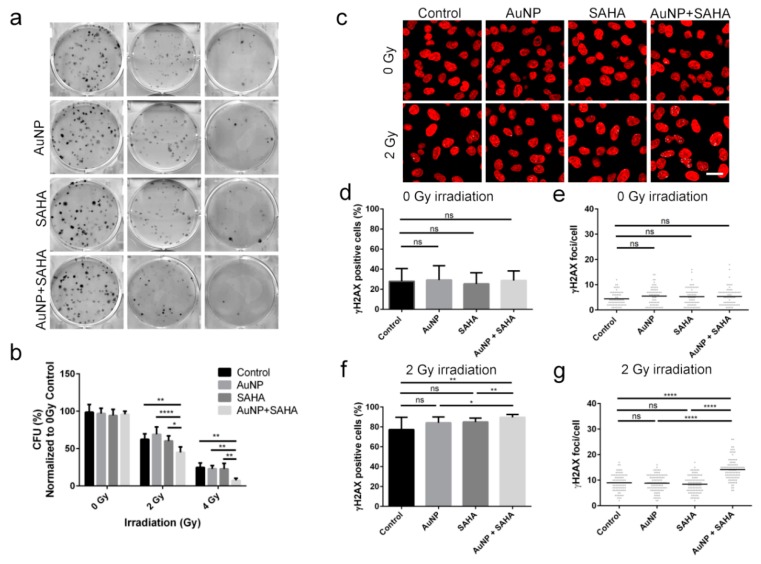
Radiosensitizing effect of AuNP and SAHA double treatments on A549 cells. (**a**) Representative pictures of the colonies of A549 cells upon AuNP, SAHA and AuNP + SAHA treatments after 0, 2 and 4 Gy irradiation. (**b**) The colony forming capacity of A549 cells was significantly lower after the combinational treatments than in the untreated and in the AuNP- or SAHA-treated samples after 2 and 4 Gy dose irradiation. The applied concentrations of AuNP and SAHA did not affect the colony forming capability of A549 cells without irradiation (* *p* value < 0.05; ** *p* value < 0.01; **** *p* value < 0.0001; two-way ANOVA Tukey’s multiple comparisons test). (**c**) Representative confocal microscopy images of the γH2AX-stained non-irradiated and irradiated A549 cells upon AuNP, SAHA and AuNP + SAHA treatments. (**d**) No differences were observed in the number of γH2AX-positive cells (**e**) or in the γH2AX foci/positive cells upon AuNP, SAHA and AuNP + SAHA treatments after 0 Gy irradiation. (**f**) The percentage of γH2AX-positive cells and (**g**) the number of γH2AX foci counted in the positively stained cells were significantly higher after AuNP + SAHA double treatments compared to the control and to the individual treatments after 2 Gy irradiation. Scale bar represents 20 µm. (* *p* value = 0.0163; ** *p* value < 0.01; **** *p* value < 0.0001 Unpaired *t*-test).

Using clonogenic assay, we can assess cell reproductive death after treatment with ionizing radiation and it can be used to determine the effectiveness of cytotoxic agents. In order to examine whether AuNPs or SAHA alone or in combination enhance the potency of irradiation, A549 cells were treated with non-toxic concentrations of AuNPs or/and SAHA and received 0, 2 and 4 Gy radiation doses, and then the colony forming capability of the samples were determined (Figure 5a,b). Both individual and combinational treatments without irradiation had no long-term effects on the colony formation ability of tumor cells. Furthermore, neither AuNP nor SAHA alone in low concentration affected the colony forming capacity of A549 cells after 2 and 4 Gy irradiation. On the other hand, combinational treatments with AuNP and SAHA followed by 2 Gy or 4 Gy radiation significantly reduced the fraction of cells, which retained the capability to form colonies compared both to the irradiated untreated samples and to the irradiated AuNP- or SAHA-treated cells as well (Figure 5a,b). 

If AuNP and SAHA together augment the cellular damage caused by ionizing radiation, the amount of DNA double strand breaks should also increase due to the combinational treatment. Therefore, A549 cells were treated either with AuNP or SAHA, or with their combination, and after receiving 0 or 2 Gy irradiation doses cells were stained to detect γH2AX loci. In order to precisely quantify the degree of DNA damage, two types of image analyses have been performed. First the percentage of γH2AX-positive cells was determined to reveal the size of affected cell population by irradiation. Secondly, in order to estimate the degree of damage caused by irradiation within the affected cell population we quantified the number of γH2AX foci/positive cells. Although the number of γH2AX-positive cells reached 80–90% upon 2 Gy irradiation in all treatment conditions (Figure 5c–f), strikingly, the incidence of γH2AX foci was significantly higher only in the case of AuNP + SAHA-treated 2 Gy irradiated samples (Figure 5c–g). On the other hand, no difference was detected in the number of γH2AX foci between the 2 Gy dose irradiated control and the 2 Gy dose irradiated, AuNP- or SAHA-treated A549 samples (Figure 5c–g), confirming the results obtained with the clonogenic assay (Figure 5a,b). Notably, without irradiation, no differences were found between non-treated and AuNP-, SAHA- or AuNP + SAHA-treated samples in the γH2AX staining, indicating that the applied combinational treatment without irradiation do not induce any DNA damage in the cells (Figure 5c–e). Our findings show that fairly radioresistant cancer cells, such as A549 cells, can be efficiently eliminated by radiotherapy following previous exposures to gold nanoparticles and the histone deacetylase inhibitor SAHA, due to the intensified DNA damage resulting from the combinational treatment approach. 

**Figure 6:**
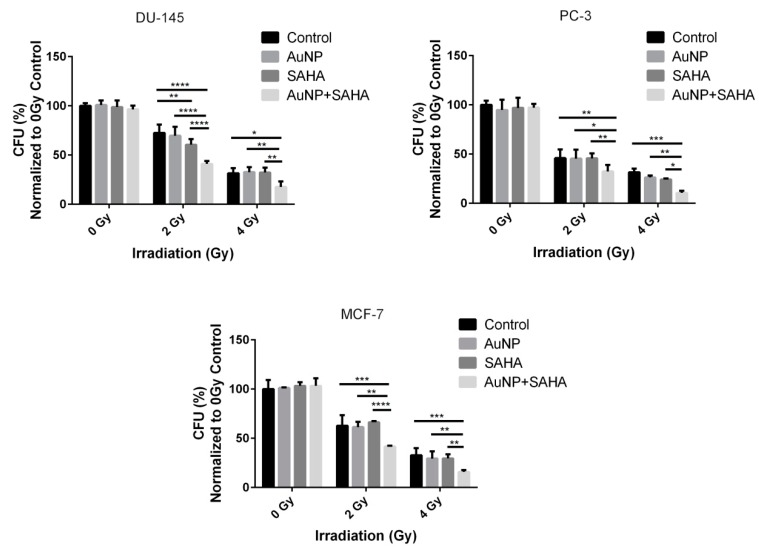
The combinational treatments affect the colony forming capacity of cancer cells upon irradiation. The AuNP + SAHA double treatments significantly decreased the colony forming capabilities of DU-145, PC-3 and MCF-7 cells after 2 and 4 Gy dose irradiation. (* *p* value < 0.05; ** *p* value < 0.01; *** *p* value < 0.001; **** *p* value < 0.0001; two-way ANOVA Tukey’s multiple comparisons test).

To test that the observed radiation enhancing features of AuNPs and SAHA manifest also on other cell types, the combinational effects of AuNPs, SAHA and irradiation were determined on a panel of different cancer cell lines, namely on DU-145, PC-3 prostate cancer and on MCF-7 breast cancer cells (Figure 6 and Supplementary Figure S2). The exact same concentrations of AuNPs and SAHA were used on these cell lines as on A549, and the irradiation was carried out with the same 0, 2 and 4 Gy doses. As expected, the AuNP + SAHA combinational treatments decreased significantly the colony forming capacity of irradiated cancer cells, while radiation treatment after the individual exposures to either AuNPs or SAHA in the applied concentrations could not attenuate the colony formation except for SAHA-treated 2 Gy dose receiving DU-145 cells (Figure 6 and Supplementary Figure S2).

**Figure 7:**
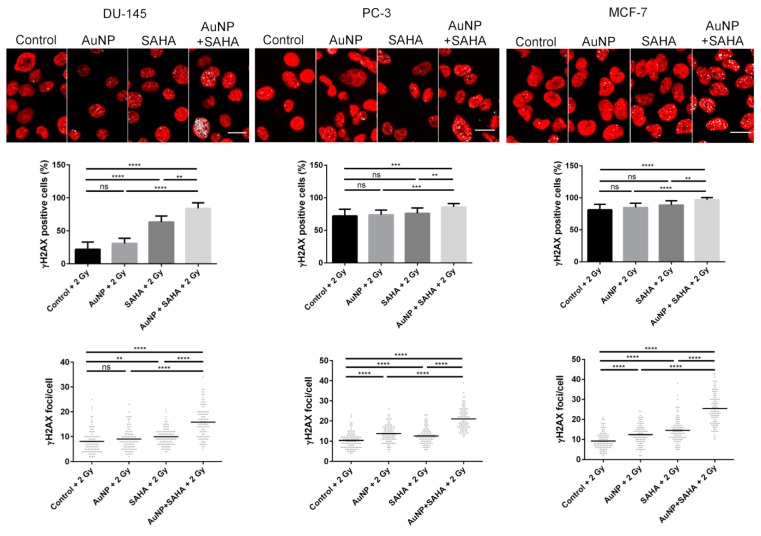
AuNP and SAHA combination induces significant DNA damage after 2 Gy irradiation. Significantly more γH2AX-positive cells were counted in the AuNP + SAHA-treated samples compared to the control and to the individual treatments after 2 Gy irradiation in all the tested cell lines. (** *p* value < 0.005 *** *p* value < 0.001; **** *p* value < 0.0001; Unpaired t-test)**.** The number of γH2AX foci was significantly increased after AuNP + SAHA treatments than in the untreated and in AuNP- or SAHA-treated γH2AX-positive cells. Scale bar represents 20 µm. (** *p* value = 0.0019; **** *p* value < 0.0001; Unpaired *t*-test).

Similar to A549 cells, the DNA damage induced by AuNP, SAHA, and irradiation was examined in DU-145, PC-3 and MCF-7 cell lines. Exposure of DU-145 cells to AuNP and radiation treatment of 2 Gy dose did not elevate the percentage of γH2AX-positive cells and the number of γH2AX foci counted in the γH2AX-positive cells, while irradiation after SAHA treatment increased significantly the amount of DNA double-strand breaks. As expected, the combinational treatment of AuNP, SAHA and irradiation caused the most significant DNA damage compared to the individual treatments. In MCF-7 and PC-3 cells individual treatments did not affect the percentage of γH2AX-positive cells in the samples, while both individual as well as AuNP + SAHA combinational treatments raised significantly the number of γH2AX foci after irradiation. However, the strongest damaging effect of the ionizing radiation was detected upon combined administration of gold nanoparticles and the HDAC inhibitor (Figure 7). In summary, the radiation-enhancing activity of the AuNP + SAHA combination is displayed in all the examined cancer cell lines. 

### 5.5. Combinational Treatment Enhances Radiosensitivity of Tumor Cells in 3D 

**Figure 8:**
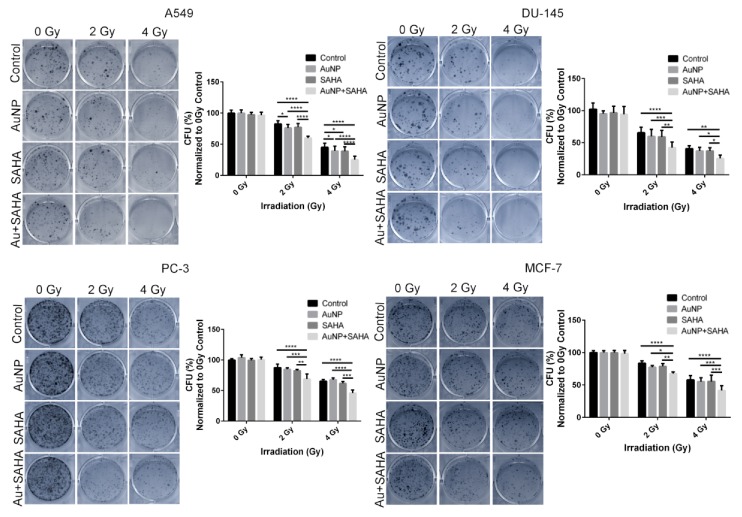
Radiosensitizing effect of AuNP and SAHA double treatments on cancer cells growing in 3D. The combination of AuNPs and SAHA significantly decreased the colony forming capability of A549, DU-145, PC-3 and MCF-7 cells in 3D cell cultures after 2 and 4 Gy irradiation compared to the control and to the individual treatments. (* *p* value < 0.05; ** *p* value < 0.01; *** *p* value < 0.001; **** *p* value < 0.0001; two-way ANOVA Tukey’s multiple comparisons test).

It is well known that 3D cell cultures often exhibit increased radio-, and chemoresistance due to cell adhesion-mediated processes [53,58]. As anticipated, for the 3D cell culture experiments the applied AuNP and SAHA concentrations had to be increased compared to those applied on 2D cultures. Without irradiation, the colony forming capability of all the tested cell lines did not change after treatment with AuNP or SAHA or their combination. However, after 2 Gy and 4 Gy irradiation the AuNP + SAHA double treatment significantly decreased the colony numbers of A549, DU-145, MCF-7 and PC-3 cells compared to the irradiated control samples and to the individual treatments as well. In case of the A549 cells after 2 Gy irradiation gold nanoparticles decreased significantly the colony forming capacity of the cells, moreover, after 4 Gy irradiation both individual treatments were effective compared to the 4 Gy irradiated untreated samples. The strongest effect on colony forming was observed when 3D cell cultures received AuNP + SAHA combinational treatments prior to irradiation (Figure 8).

**Figure 9:**
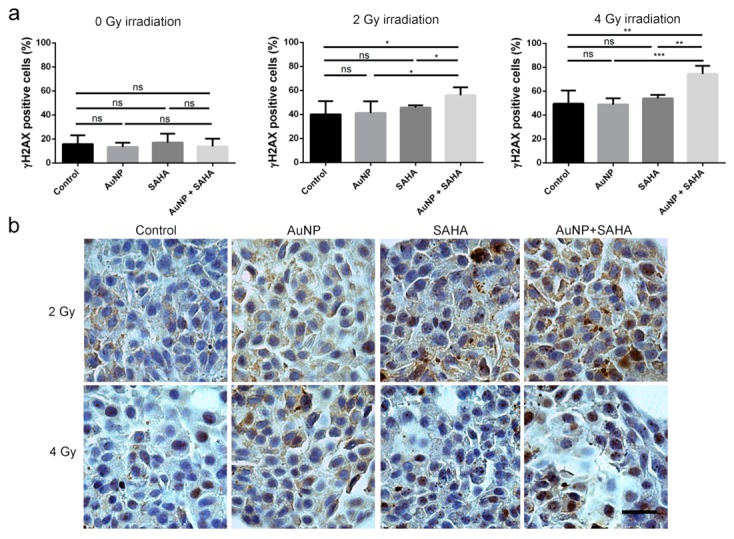
AuNP and SAHA treatments enhance the DNA damaging effect of irradiation in A549 3D cell cultures. (**a**) The percentage of γH2AX-positive cells in the samples irradiated with 2 and 4 Gy doses were significantly higher after the double treatments than in the control and in the AuNP- or SAHA-treated samples. No differences were observed in the non-irradiated AuNP-, SAHA- or AuNP + SAHA-treated cells. (b) Representative images of γH2AX-stained untreated and AuNP-, SAHA- or AuNP + SAHA-treated A549 spheroids after 2 Gy and 4 Gy irradiation. Scale bar represents 100 µm. (* *p* value < 0.05; ** *p* value < 0.01; *** *p* value = 0.0009; Unpaired *t*-test).

We detected the extent of DNA damage induced by chemo- and radiation therapy in 3D cancer cell spheroids by γH2AX staining. Compared to the untreated samples as well as to the spheroids receiving individual treatments of either AuNP or SAHA, significantly more DNA damage was observed in the AuNP + SAHA-treated A549 spheroids after irradiation using 2 Gy and 4 Gy doses. After 2 Gy irradiation, 59% of the total cell number was γH2AX-positive in AuNP and SAHA double treated samples, while in the control and in AuNP- or SAHA-treated spheroids 41–47% γH2AX-positive cells were counted. In the 4 Gy irradiated spheroids the combinational treatments resulted in increased percentage of γH2AX-positive cells (76%), which was significantly higher than the percentage measured in the control and in the individual AuNP- or SAHA-treated samples (50–55%) (Figure 9).

## 6. Discussion

Radiotherapy, employed either alone or in combination with surgery, chemo- or immunotherapy, represents one of the leading strategies for the treatment of primary and metastatic tumors [59,60]. Although the radiation applied in modern, state-of-the-art therapeutic modalities are image-guided and intensity-modulated, nevertheless, it is fraught with limitations [61,62]. These drawbacks encompass inherent or acquired radioresistance which is frequently observed in certain breast, non-small cell lung and androgen-independent prostate cancers. To overcome such radioresistance an increased irradiation dose should be applied, ultimately leading to the undesired consequences of damaging healthy organs and tissues in the anatomical proximity of the targeted tumor [63,64,65,66,67,68]. Therefore, in recent years great impetus has been gained in developing and optimizing various types of materials for radiation-enhancing purposes in order to avoid the employment of excessisve radiation doses to eradicate cancer and simultaniously to protect healthy tissues. The clinical translation of such radiosensitizers could open novel avenues in oncotherapeutical approaches particularly for the treatment of radioresistant tumors. Numerous small-molecular drugs were identified as potential radiosensitizers, targeting distinct features of the tumor on the subcellular, cellular and tissue-level such as modification of chromatin organization, inhibition of DNA damage response, targeted inhibition of cell cycle checkpoint machinery or modulation of the tumor microenvironment [69,70]. 

Chromatin organization is tightly regulated by covalent modifications of core histones. Maintaining high levels of acetylated histones by regulating the activity of histone deacetylase enzymes neutralizes the positive charge on lysine residues, which leads to a massive reduction in the interactions between the negatively charged genomic DNA and histone proteins [38]. This fairly relaxed, open chromatin structure is highly accessible to transcription factors and enables active gene expression, albeit rendering the DNA more susceptible to various damaging factors [49,71]. Importantly, several pharmacological inhibitors have been developed to enhance histone acetylation by targeting HDAC enzymes. Among these, the application of romidepsin and SAHA is already approved in clinical practice, demonstrating their relevance in therapeutic modalities [72]. Nevertheless, inducing chromatin relaxation by histone-hyperacetylation holds enormous relevance in the context of radiation enhancement as well, since irradiation-induced reactive electrons and the concomitantly generated free radicals can ultimately target the DNA and such ionization events produce DNA base damage, DNA single strand breaks and double strand breaks [33,73]. 

An increasing body of literature indicated that in addition to small-molecular anti-cancer compounds, metal nanoparticles can also be employed to preferentially sensitize tumor cells to ionizing radiation [13,74]. Nanoparticle-based treatment modalities exploit the unique characteristics of the tumor physiology, such as the poor lymphatic drainage and the leaky vasculature leading to enhanced retention and accumulation of nanomaterials in the tumor microenvironment [75]. It has been reported that gold nanoparticles are biologically inert, and therefore exhibit excellent biocompatibility [23]. However, when irradiated, gold nanoparticles accumulated in the tumor tissue are able to amplify the efficiency of irradiation, thus, displaying an excellent radiosensitizing effect [24]. In addition to this, as was shown in pancreatic cancer models, AuNPs interact with stromal cells and affect the cancer cell-tumor stroma communication via suppressing the expression of several autocrine and paracrine sinaling factors and inhibit angiogenesis, further demonstrating their feasibility in cancer therapy [76,77,78]. 

Based on the unique features of HDAC inhibitors and gold nanoparticles, we hypothesized that the HDAC inhibitor SAHA could further potentiate the radiosensitizing action of AuNPs and vice versa, AuNPs can increase the radio-enhancing features of SAHA, resulting in a synergistically augmented tumor eradication potency of combinational cancer therapy. More precisely, our concept was to prove that the mechanism of action of AuNPs and those of SAHA complement each other since SAHA renders the cells more susceptible to damaging agents while AuNPs multiply the amount of reactive particles generated by irradiation culminating in a synergistic radio-enhancement. Therefore, we prepared citrate-coated AuNPs of approximately 10 nm diameter and tested their combinational action with SAHA on various irradiated 2D cancer cell cultures as well as 3D spheroids. Cancer cells internalized the nanoparticles, which accumulated mainly in the cytoplasm or appeared in multilamellar bodies. The amount of acetylated histones indicated that intracellular AuNPs did not interfere with the HDAC-inhibiting activity of SAHA. Calculated combinational indices proved that the nature of the combined radiosensitizing effect of AuNPs and SAHA is in fact synergistic and not simply additive. Attenuation of the colony forming ability of cancer cells and the increased amount of DNA double strand breaks within cell nuclei also verified the effective potentiation of the radio-enhancing capabilities of the SAHA and AuNP combination. 

Three-dimensional spheroids recapitulate much better the cellular and physiological features of actual tumor tissues than 2D cultures, owing to a correct spatial architecture, cell polarity, anchorage-independent growth and also due to proper cell-cell and cell-matrix interactions in the 3D configuration. Moreover, spheroids reflect hypoxia and metabolic gradients as well as radio- or drug-resistance analogously to patient-residing solid tumors [53,79]. Thus, for the accurate assessment of biological performance of therapeutic drugs, it is essential to screen their efficiency not only on 2D monolayers but on 3D platforms as well. Considering these facts, we tested the anti-cancer efficacy and radiosensitizing features of AuNP + SAHA combination also on homotypic 3D spheroids, prepared of four different cancer cell lines, among them radioresistance displaying A549 and DU-145 cancer cells. Although our current knowledge on histone acetylation in tumor tissues is largely scarce, in a previous study the authors claimed hypoacetylated histones and highly condensed chromatin in cells grown in 3D, raising the issue of whether elevated levels of heterochromatin in 3D cultured cells and in solid tumors might protect them from the severe effects of ionizing radiation [56]. Considering this information, modulation of chromatin structure by HDAC inhibitors might be a viable and efficient approach to attenuate radioresistance. It has been reported that cells grown in 3D exhibit higher radioresistance than in 2D monolayers, indicating that cellular attachment to extracellular matrix components, such as fibronectin or laminin, as well as cellular shape and morphology (flat or round-shaped) might also be responsible at least in part for the variation in radiation tolerance and survival [52,79]. Indeed, we also observed that the applied concentrations of AuNP and SAHA had to be optimized and set somewhat higher (from 6.8 µM to 34 µM AuNP and from 0.1 µM to 0.5 µM or 1 µM SAHA) in 3D spheroids compared to 2D cultures. Interestingly, cancer cells grown within 3D spheroids, receiving radiotreatment, produced more colonies upon subsequent colony forming assays than cells in a 2D set-up. These results imply first of all that those cells in the spheroids that survive irradiation gain a certain selection and growth advantage and, secondly, that cells grown in 3D intrinsically bear lower sensitivity to irradiation. Nevertheless, exposure of cancer spheroids to AuNP + SAHA combination prior to radiotreatment, significantly increased the amount of DNA double strand breaks and reduced the number of colonies. This observation proves that the SAHA-triggered chromatin relaxation complemented by the AuNP-mediated multiplication of irradiation manifests also in 3D spheroids validating our concept and further demonstrating the synergy between gold nanoparticles and HDAC inhibitors upon irradiation in a more realistic configuration.

In summary, we found that SAHA treatment facilitated the irradiation-induced DNA damaging potential of AuNPs. The observed synergistic radiation-enhancing feature can be attributed to the SAHA-mediated relaxation of the chromatin structure. We believe that novel combination regimens of metal nanosystems and HDAC inhibitors such as AuNP and SAHA represent a highly versatile platform for next-generation radiosensitizing nanomedicines that can be exploited to attenuate cancer cell growth for an improved therapeutic outcome.

## Supplementary Materials

The following are available online at https://www.mdpi.com/2079-4991/10/1/158/s1, Figure S1: The effect of AuNP, SAHA and the combination of AuNP and SAHA on the viability of cancer cells after irradiation. Figure S2: The AuNP + SAHA double treatment affects the colony formation of cancer cells upon irradiation.
