# Supplementary material for: Synergistic Radiosensitization by Gold Nanoparticles and the Histone Deacetylase Inhibitor SAHA in 2D and 3D Cancer Cell Cultures
Source: Nanomaterials (Basel). 2020 Jan 16;10(1):158. doi: 10.3390/nano10010158 (PMC7023030; doi:10.3390/nano10010158)
Supplement: Supplementary file 1 [file nanomaterials-10-00158-s001.pdf]

Supplementary Materials

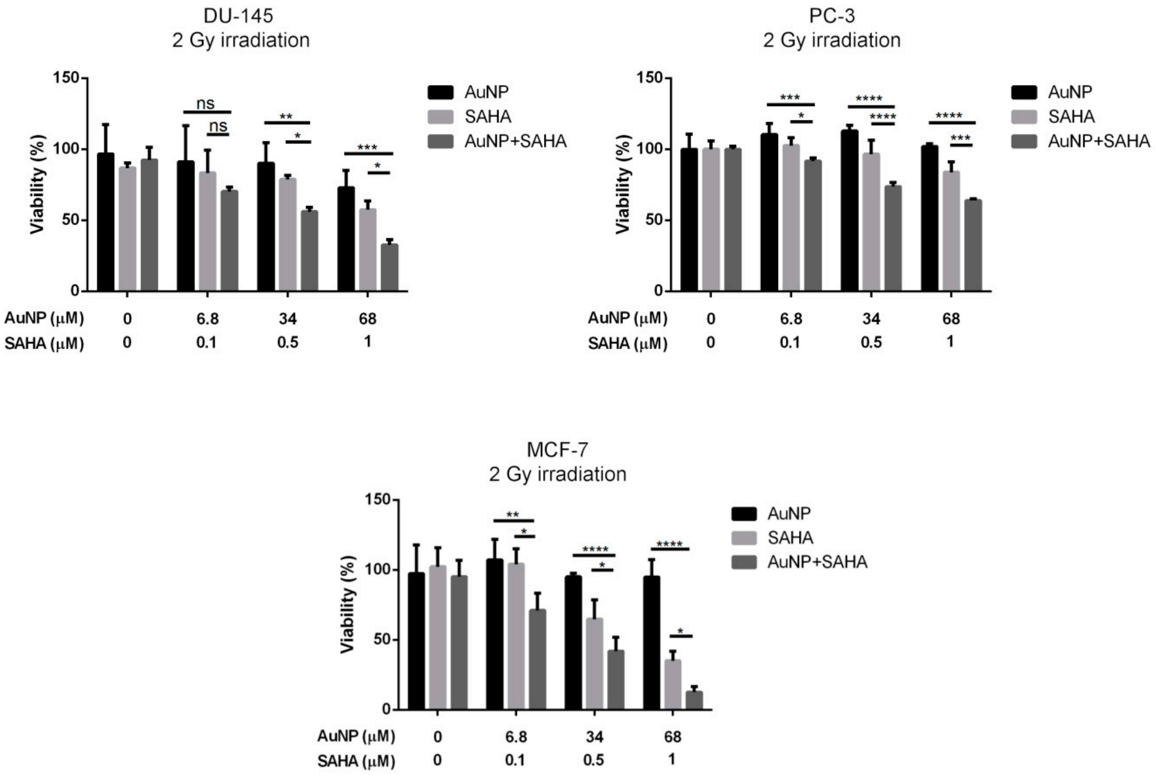

Supplementary Figure 1. The effect of AuNP, SAHA and the combination of AuNP and SAHA on the viability of cancer cells after irradiation. In the applied concentrations the double treatments were the most effective in all the tested cell lines. On DU-145 cells AuNPs and SAHA double treatment in low concentration did not affect significantly the cell viability, but in higher concentrations similarly to PC-3 and MCF-7 cells AuNP+SAHA combinational treatments significantly decreased the cell viability compared to the control and to the individual treatments after 2 Gy irradiation. (*P value < 0.05; **P value < 0.01; ***P value < 0.001; ****P value < 0.0001; Two-way ANOVA Tukey's multiple comparisons test)

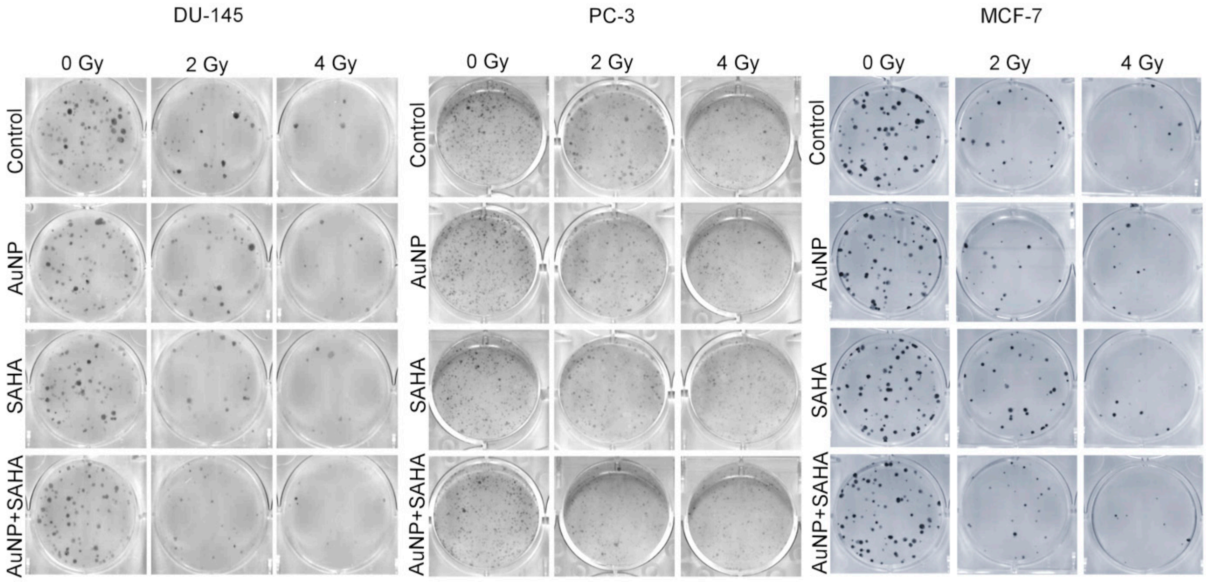

Supplementary Figure 2. The AuNP+SAHA double treatment affects the colony formation of cancer cells upon irradiation. Lower number of DU-145, PC-3 and MCF-7 cancer cell colonies were observed upon AuNP+SAHA double treatments after 2 and 4 Gy dose irradiation than in the untreated or in the AuNP- or SAHA-treated samples.
